# Additive Manufacturing in Orthopaedic Trauma: Current Evidence and Applications

**DOI:** 10.3390/medicina62030599

**Published:** 2026-03-21

**Authors:** Nikolaos A. Stavropoulos, Fotios Kantas, Dimitrios V. Papadopoulos, Vasileios S. Nikolaou, George C. Babis

**Affiliations:** 1 Second Department of Orthopaedic Surgery, School of Medicine, National and Kapodistrian University of Athens, “Konstantopouleio” General Hospital, 14233 Athens, Greecegeorge.babis@gmail.com (G.C.B.); 2 School of Medicine, National and Kapodistrian University of Athens, 11527 Athens, Greece; f.d.kantas@gmail.com

**Keywords:** additive manufacturing, 3D-printed anatomical models, 3D-printed patient-specific surgical guides, 3D-printed implants, 3D-printed external fixators

## Abstract

Additive manufacturing also known as three-dimensional printing (3D printing), provided the ability to produce precise three-dimensional structures, representing a rapidly growing field in Orthopaedics. Its clinical value has been attributed to the ability to create complex three dimensional objects with relative ease and at low cost. However, the available evidence regarding its applications in trauma was heterogeneous. This narrative review aimed to analyze the clinical applications of 3D printing in traumatology. Additionally, the research gaps that emerged in our literature search were underscored. Four application domains were selected based on their prevalence in the screened literature and relative level of clinical implementation within orthopaedic traumatology, including (1) 3D-printed anatomical models, (2) patient-specific surgical guides (PSSGs), (3) 3D-printed implants, and (4) temporary 3D-printed external fixation devices. 3D-printed anatomical models were found to help in reducing operative time, estimated blood loss, and the intraoperative radiation exposure. The use of PSSGs was shown to improve intraoperative accuracy and to provide a basis for consistent, accurate, and reproducible outcomes. However, their implementation was hindered by preparation time, the need for stable anatomical landmarks, and reduced accuracy due to potential soft-tissue injury and swelling. In contrast, 3D-printed implants and external fixation devices constituted promising but less extensively studied applications of 3D printing in trauma. The production of customized implants and external fixators, as suggested by the studies available, was deemed feasible, with comparable mechanical properties and significantly lower cost. Larger multicenter studies are required to support and validate these findings. Overall, based on the available evidence, 3D-printed anatomical models and patient-specific surgical guides demonstrate the highest level of clinical applicability, primarily in preoperative planning and intraoperative guidance.

## 1. Introduction

Additive manufacturing, commonly known as 3D printing, allows for the creation of three-dimensional objects layer by layer. Its benefits include design freedom, complex shape formation, wide range of suitable materials and fast production [1]. Additive manufacturing’s advantages over conventional production techniques carry over in orthopaedics with increasing volume of research in the field utilizing 3D printing. Most high-quality evidence and standardized workflows for 3D printing stem from elective orthopaedic procedures, especially adult reconstruction, while trauma-specific research remains heterogeneous and less standardized [2]. Recent systematic reviews in elective orthopaedic surgery have demonstrated the increasing integration of additive manufacturing in patient-specific implant design and surgical planning, although the lack of standardized outcome measures continues to limit inter-study comparability. Similarly, contemporary review studies have highlighted the expanding role of 3D printing in personalized orthopedic care, including patient-specific anatomical modeling and customized implant design; however, trauma-specific applications remain less consistently investigated and lack standardized clinical workflows [3]. The most frequent application of 3D printing in trauma settings is preoperative planning and patient specific surgical guides (PSSGs) [4]. Traditional approaches to preoperative planning incorporate radiography, Computed Tomography (CT) imaging, and occasionally physical models. The standard approach provides limited intuitive three-dimensional information about the patient’s unique anatomy. Moreover, there is no reliable haptic and spatial feedback. Additive manufacturing enables the efficient production of patient-specific anatomical models. Digital Imaging and Communications in Medicine (DICOM) data are transformed into a physical form accurately replicating the patient’s anatomy [5]. In addition to improved visualization and depth perception, they enable surgeons to better understand fracture morphology and allow for a preoperative simulation [4].

Beyond 3D-printed anatomical models, 3D printing has expanded into intraoperative applications, particularly through patient-specific surgical guides. PSSGs represent an increasingly researched application of 3D printing allowing for accurate reproduction of preoperatively planned surgical parameters such as planned screw placement, length, and trajectory as well as osteotomy range and angle [6]. In addition, they provide a position-independent solution, increasing surgeon confidence and potentially shortening the learning curve for surgeons [7,8,9]. Overall, PSSGs represent a promising tool potentially achieving quicker and more accurate procedures while reducing fluoroscopy requirements [9].

Additive manufacturing offers the ability to produce 3D-printed surgical implants matching the patient’s unique anatomy. Surgical implants constitute a rapidly growing area of use [4]. Most of the available research regarding 3D-printed surgical implants has focused on their use in elective orthopaedic procedures, mainly arthroplasties. In trauma, manufacturing timelines and regulatory requirements have limited their use in post-traumatic segmental bone loss, deformity, and failed fixation.

3D-printed external fixators are an experimental application of 3D printing in trauma surgery. Conventional external fixators are expensive and may not always surprisingly be easily accessible [10]. Primary objective of research on 3D-printed external fixators seems to emphasize on their potential mechanical features and significant cost imbalance. The production time is also a matter to consider.

**Figure 1:**
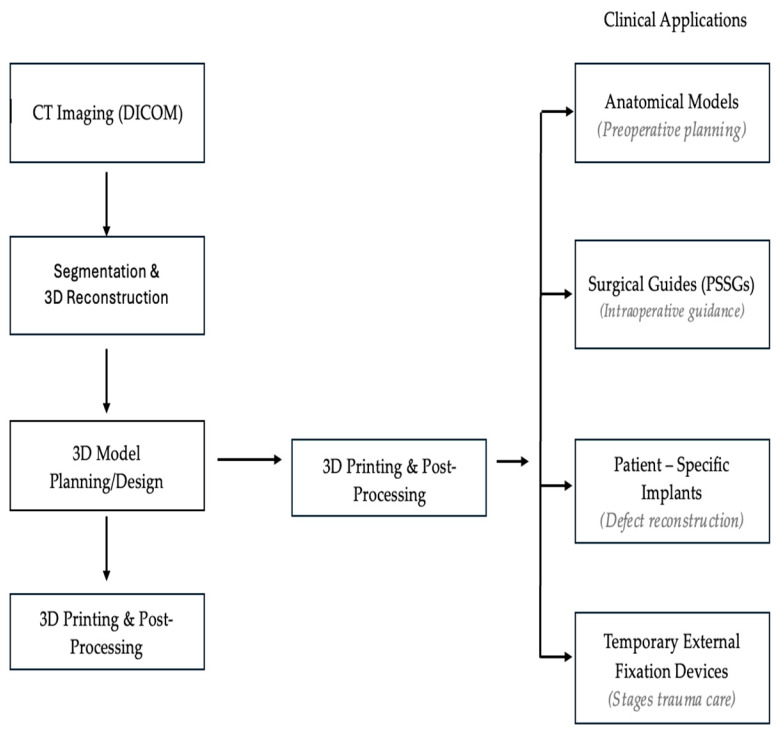
Workflow of clinical 3D printing in orthopaedic trauma, from CT imaging and segmentation to 3D model design, and clinical applications.

This narrative review provides an overview of current evidence regarding applications of 3D printing in orthopaedic trauma. Moreover, the core findings in four unique areas will be discussed: 3D-printed anatomical models, PSSGs, 3D-printed implants, 3D-printed temporary external fixators Figure 1.

## 2. Methods

### 2.1. Study Design

A narrative review aiming to summarize current evidence regarding the clinical applications of three-dimensional (3D) printing in orthopaedic trauma surgery was conducted. We focused on four main domains: (1) 3D-printed anatomical models for preoperative planning, (2) patient-specific surgical guides (PSSGs), (3) patient-specific 3D-printed implants, and (4) temporary 3D-printed external fixation constructs. A structured literature search was conducted to improve transparency of study identification and selection.

### 2.2. Literature Search Strategy

A targeted literature search was performed using the electronic databases PubMed/MEDLINE and Scopus to identify relevant studies published up to December 2025. Studies published between January 2020 and December 2025 were considered eligible in order to reflext contemporary clinical applications of additive manufacturing in orthopaedic trauma. The search included a combination of terms and keywords related to additive manufacturing and trauma surgery, including: “*3D printing*”, “*three-dimensional printing*”, “*additive manufacturing*”, “*orthopaedic trauma*”, “*fracture*”, “*anatomical model*”, “*patient-specific surgical guide*”, “*template*”, “*custom implant*”, and “*external fixator*”. Moreover, through manually screening reference lists of selected articles and relevant reviews, additional references were identified.

### 2.3. Eligibility Criteria

Publications were considered eligible if they evaluated or reported clinically relevant aspects of 3D printing in orthopaedic trauma care, including feasibility, accuracy, operative parameters, fluoroscopy use, complication profile, or reconstructive outcomes in post-traumatic bone defects. Clinical studies, cadaveric investigations, and biomechanical or technical studies were included only when directly applicable to trauma surgery. Articles focusing exclusively on elective orthopaedic procedures without trauma relevance were excluded unless they provided transferable, trauma applicable, insights regarding workflow or surgical guide performance. Full-text articles were excluded if they focused exclusively on elective orthopaedic procedures without trauma relevance, engineering or materials-testing applications without clinical context, or constituted review articles, editorials, or conference abstracts lacking primary data.

### 2.4. Study Selection and Data Extraction

**Figure 2:**
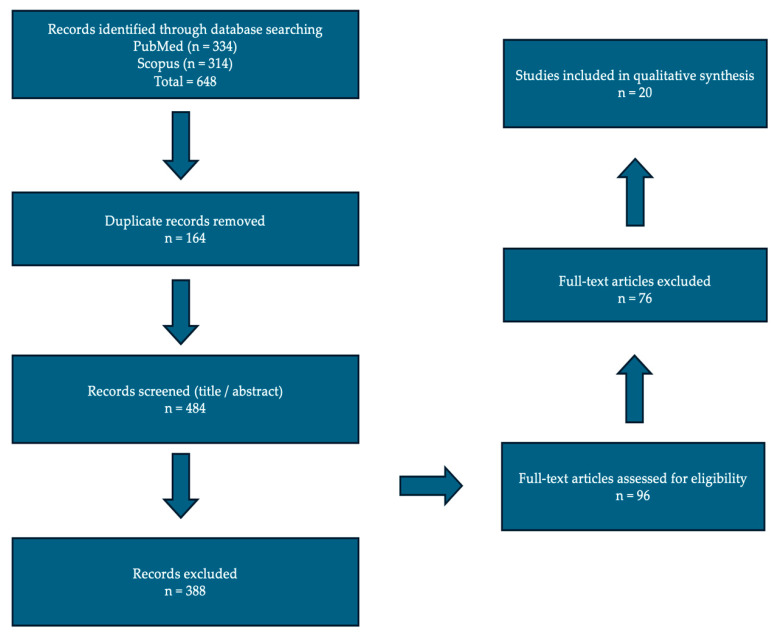
A simplified flow diagram depicting the selection review process.

Titles and abstracts were screened for relevance, followed by full-text assessment of eligible articles. Duplicate records were identified and removed prior to screening. Extracted data included study design, anatomical region, trauma indication, type of 3D-printing application, workflow characteristics (imaging modality, planning/segmentation steps, production time when available), and reported outcomes (e.g., accuracy metrics, operative time, fluoroscopy exposure, blood loss, complications, and functional measures). A simplified flow diagram of the study identification and selection process is provided in Figure 2.

## 3. Results

### 3.1. 3D-Printed Anatomical Models

Additive manufacturing has been evaluated in clinical studies involving complex articular fractures, joint reconstructions or inaccessible injuries [11,12,13]. The visualization, depth perception, haptic feedback attained through 3D-printed models have been shown to increase junior and mid-level surgeon confidence. Moreover, senior surgeons also report a positive effect in decision making and surgical approach [14]. The involvement of 3D-printed anatomical models in surgical planning has been shown to consistently reduce operative time, estimated blood loss, and radiation exposure [11,12]. In elderly patients with complex proximal humeral fractures, the use of 3D-printed models was associated with reduced operative time and intraoperative blood loss in a comparative clinical study [12]. In high-energy tibial plateau fractures, the use of patient-specific 3D-printed models was associated with improved preoperative planning and reduced operative time in a comparative clinical study [15]. Similar findings were described in observational trauma cohorts using patient-specific fracture models [11]. Reduced intraoperative fluoroscopy use has been reported in comparative clinical trauma studies using patient-specific 3D-printed fracture models for preoperative planning [16].

Manufacturing cost remains institution dependent. Preparation time may be considered as a burden in Trauma practice. The development of a patient-specific anatomical model typically requires high-resolution imaging, image segmentation, computational modelling, and 3D printing [4]. The reported preparation time varies from approximately 5 to 72 h [14,17]. Nevertheless, ongoing advances in 3D-printing technology indicate a decreasing trend in overall preparation time [5,18].

### 3.2. Patient-Specific Surgical Guides

Patient-specific surgical guides (PSSGs) enable the translation of a digitally planned trajectory into the operating field. PSSGs have been reported to enhance fixation accuracy and improve screw positioning by ensuring controlled and reproducible entry points and trajectories [7,19,20,21]. More specifically, clinical studies comparing postoperative results with preoperative planning have demonstrated improved alignment and accuracy when guide-assisted techniques were used [7,19,20]. Furthermore, PSSGs facilitate preoperative determination of the screw length and direction, thereby reducing the need for intraoperative adjustments [7,19] and minimizing intraoperative fluoroscopy use in pelvic and sacroiliac fixation procedures [7,20].

In trauma-specific applications, a retrospective clinical cohort study of distal femoral fractures reported smaller postoperative alignment differences and absence of postoperative deformity in the navigation-template group compared to conventional fixation, while functional outcomes as measured by KSS and FKSS remain unchanged [19]. In clinical studies assessing sacroiliac screw placement, guide-assisted techniques were associated with improved positioning accuracy and reduced fluoroscopy use [7,20]. They also facilitate first-attempt guidewire placement in percutaneous scaphoid fixation and allow accurate screw direction with improved reduction metrics in tibial plateau fractures [8,9]. A clinical feasibility study evaluating guide-assisted pin placement for external fixation reported technical applicability and reproducible positioning in clinical settings [22]. Collectively, these findings showcase that 3D-printed PSSGs enhance fixation accuracy, improve workflow efficiency, and contribute to better anatomical alignment in complex trauma cases.

### 3.3. 3D-Printed Implants

In excessive bone loss, 3D-printed implants have been used in clinical settings to provide patient-specific reconstruction of segmental defects. Titanium alloys are commonly utilized materials for additively manufactured implants (especially Ti6Al4V) [23]. On the other hand, polymer-based materials are often used for anatomical models or surgical guides [24]. Titanium mesh structures exhibit porous architectures in which pore size and porosity are designed to facilitate bone ingrowth, as described in comparative implant analyses [23].

In trauma and revision settings, retrospective clinical series have reported the use of custom 3D-printed porous titanium implants for the management of critical-sized bone defects [25]. Radiographic implant integration and maintained construct stability were reported during follow-up. At a minimum one-year follow-up, 74% of patients did not require subsequent surgical intervention [25]. In a biomechanical study, 3D-printed locking screws maintained their mechanical features which were comparable to those of machined or hand-tapped ones for definite fixation [26]. Clinical series have reported favorable outcomes with the use of 3D-printed cones and sleeves for severe bone defects compared to conventional ones demonstrating primary stability and anatomical restoration [27]. Outcomes support the clinical application and relevance of patient-specific 3D-printed implants in trauma, particularly in complex cases, demonstrating their ability to provide stable fixation, bone healing, and function restoration in cases of severe post-traumatic bone loss.

### 3.4. Temporary 3D-Printed External Fixation Devices

In trauma, temporary external fixation is commonly applied in open type fractures or in polytrauma cases where Damage Control is undertaken [28]. Evidence regarding 3D-printed temporary external fixation devices is predominantly limited to experimental and preclinical studies. Published reports include design-development studies and in vitro mechanical testing evaluating construct stiffness, stability, and feasibility of application [29,30].

Mechanical analyses comparing novel 3D-printed external fixator designs with industry-standard devices have demonstrated comparable construct stiffness under laboratory conditions [31]. Reported production costs for certain 3D-printed designs were lower than those of commercially available systems [29,30]. Design studies have described the feasibility of producing external fixation components using additive manufacturing, including the potential for templated pin positioning [30]. A clinical feasibility study assessing guide-assisted external fixator pin placement reported accurate and reproducible positioning in patients undergoing pelvic fixation [22].

Manufacturing time for certain 3D-printed external fixation designs has been reported to be less than one hour under controlled conditions [30]. However, no large-scale clinical outcome studies evaluating 3D-printed temporary external fixation in acute trauma patients were identified.

Overall, these findings suggest that 3D-printed temporary fixations offer an efficient, cost-effective, mechanically sufficient, and patient-specific alternative to conventional devices, potentially improving workflow and fracture management.

The main clinical applications of 3D printing in orthopaedic trauma, along with their reported benefits and limitations, are summarized in Table 1.

## 4. Discussion

Collectively, the available evidence suggests that 3D printing technologies may enhance several aspects of orthopaedic trauma management. Among the currently investigated applications, 3D-printed anatomical models appear to represent the most established and clinically integrated use, particularly in complex fracture patterns where improved spatial understanding may facilitate preoperative planning and surgical decision-making. Patient-specific surgical guides further translate preoperative digital planning into the operative field and may improve fixation accuracy in anatomically constrained procedures requiring precise screw trajectories. Patient-specific implants have demonstrated promising applications in the reconstruction of complex post-traumatic bone defects, where customized implant geometry and porous architectures may support implant stability and bone healing. In contrast, applications involving temporary 3D-printed external fixation devices remain largely supported by biomechanical and feasibility studies, and robust clinical validation is still limited. It appears that 3D printing may serve as a valuable tool in orthopaedic practice [1,2,4]. recent trends demonstrate a shift toward exploring additional uses, especially in trauma [2,3]. The production process of 3D-printed constructs has developed rapidly over recent decades, particularly in the last few years [1,32]. A few years ago, having a 3D printer within hospital facilities was considered impractical, whereas today, many departments can be equipped with one [1,18]. Beyond acquisition costs, the entire workflow for producing a final product has been simplified and has become more accessible [5,18]. Notably, what once required several days can now be completed within hours, from trauma imaging to the production of the final 3D-printed output [11,17,31]. This acceleration has enabled broader experimentation and application in the trauma setting. To date, the largest proportion of research and the greatest integration into clinical practice involve anatomical models [4]. Anatomical models, which were occasionally used in routine preoperative planning, can now be produced based on the patient’s unique anatomy and injury pattern. Their most effective application appears to be in complex, comminuted fractures and in anatomically difficult-to-access regions. Improved preoperative understanding of the injury seems to result in reduced operative time, lower estimated blood loss, and decreased required radiation exposure [11,12,17,18]. Operative duration itself has been associated with perioperative complications in surgical populations, which further highlights the potential clinical relevance of reducing operative time in trauma procedures [33]. In addition, greater agreement has been observed among involved surgeons regarding the surgical approach, patient positioning, and overall surgical plan [11,14]. Furthermore, among less experienced surgeons, residents, and even medical students, comprehension of the surgical approach was achieved more rapidly compared with conventional planning methods. Importantly, even highly experienced trauma surgeons have recognized the contribution of these models to surgical planning [11,13,14]. In contrast to patient-specific surgical guides (PSSGs), 3D-printed implants, and 3D-printed external fixators, the integration of 3D-printed anatomical models into daily clinical practice appears more achievable; rather than altering the surgical plan, they act supplementary alongside to the conventional preoperative planning.

PSSGs are real-time trauma anatomy-based intraoperative tools [6,34]. The literature regarding their use in trauma is scattered and heterogeneous. PSSGs face the same limitation encountered with anatomical models, namely the required preparation time [5,6,18,34]. While anatomical models are primarily advisory in nature, PSSGs may modify the decisions of the surgical team. Specifically, provided that correct positioning is achieved, they can supply information such as the ideal placement, angle, and depth of screws [6,7,8,9]. Their current applications include percutaneous screw fixation and minimally invasive techniques, where even small errors in screw trajectory may have serious consequences, such as failure of fixation. In such cases, such as iliosacral screw placement, guided scaphoid fixation, and selected periarticular fractures (e.g., tibial plateau and distal femur), PSSGs have demonstrated improved accuracy and faithful adherence to the preoperative plan [7,8,9,19]. However, these data derive from studies with small patient samples, conducted across different anatomically constrained regions, and using non-standardized and variable methods of outcome assessment [34,35]. Furthermore, soft tissue injury, local swelling, and the requirement for stable anatomical landmarks limit the effective and universal reproducibility of their success. It is also worth noting that the theoretical advantage of the operative time reduction may be balanced by the additional time required for soft tissue processing [6,20,21,34]. Finally, the theoretically reduced radiation exposure may likewise be counterbalanced by the need for additional imaging to confirm successful application of the PSSG [7,19,22,34]. Beyond technical feasibility, the clinical implementation of patient-specific surgical guides and custom 3D-printed implants is also influenced by regulatory and certification requirements. In the European Union, such devices must comply with the Medical Device Regulation (MDR 2017/745), which introduces stricter requirements regarding device classification, traceability, and post-market surveillance [36]. Similarly, in the United States, patient-specific implants and surgical guides are regulated by the Food and Drug Administration (FDA), which provides dedicated guidance for additive manufactured medical devices and patient-matched devices [37]. These regulatory pathways may increase production timelines and costs and therefore represent an important practical barrier to the widespread clinical adoption of 3D printing technologies in orthopaedic trauma.

Although 3D-printed implants and 3D-printed external fixators represent the most advanced applications of 3D printing, their integration into clinical practice remains quite limited [2,4]. 3D-printed implants may offer a significant advantage in the management of excessive post-traumatic defects and segmental bone loss, through improved anatomical fit and porous structures that promote osseointegration. More specifically, they are theorized to outperform conventional implants in both biomechanical performance and osseointegration due to the high adaptability of their porous microstructure [23,24,38]. The available evidence in the literature is limited, and most studies again focus on non-urgent procedures [4,24,38]. Regarding 3D-printed external fixators, the existing literature mainly focuses on their mechanical properties and production cost. These small-sample or feasibility studies are not sufficient to draw a solid clinical conclusion. Nevertheless, the cost of 3D-printed constructs appears to be clearly lower, while their mechanical properties are comparable [29,30,39]. Three-dimensional printing should also be interpreted within the broader context of digital technologies increasingly used in orthopaedic surgery, including computer navigation, robotic-assisted surgery, and augmented reality (AR). While navigation and robotic systems primarily provide real-time intraoperative guidance, additive manufacturing mainly contributes to preoperative planning through the creation of patient-specific anatomical models, surgical guides, and implants [40,41]. Unlike navigation or robotics, 3D printing does not require complex intraoperative hardware integration but instead translates digital planning into physical tools used during surgery. These technologies should therefore be considered complementary rather than competing approaches within the evolving digital workflow of trauma surgery.

## 5. Limitations

This narrative review has several limitations. First, the available literature on 3D printing in orthopaedic trauma remains heterogeneous, with most studies consisting of small clinical cohorts, feasibility studies, or biomechanical analyses. Second, due to the narrative design of this review, the selection and interpretation of studies may be subject to selection bias. Additionally, outcome measures and reporting methods differed substantially across studies and anatomical regions, limiting direct comparison of results. Finally, the absence of standardized study designs and prospective comparative trials limits the strength of the currently available evidence.

## 6. Conclusions

Overall, 3D printing appears as an increasingly viable solution for orthopaedic trauma, with certain requirements for integration into clinical practice. 3D-printed anatomical models appear to represent the most established and clinically integrated use in trauma surgery. Patient-specific surgical guides and implants demonstrate promising results, although the available evidence remains heterogeneous. The use of 3D printing for the production of external fixators is also highly promising, although the available trauma-specific data remain very limited. Future research should aim at supporting and validating the existing clinical evidence through larger, prospective, and ideally multicenter studies.

## Figures and Tables

**Table 1:** Current clinical applications of 3D printing in orthopaedic trauma.

3D-Printing Application	Main Purpose in Trauma	Key Reported Benefits/Outcomes	Main Limitations & Barriers	Current Maturity/Evidence Strength
3D-printed anatomical models	Preoperative planning and enhanced fracture understanding (patient-specific anatomy, fracture morphology)	Improved spatial understanding and surgical planning; increased surgeon confidence; reported reductions in operative time, blood loss, and intraoperative fluoroscopy use	Preparation time required for image segmentation and model production; institutional cost variability; may not be feasible in urgent trauma cases	Most established clinical application with multiple comparative clinical trauma studies
Patient-specific surgical guides (PSSGs)	Translate digital planning into accurate intraoperative execution (e.g., screw trajectory, osteotomy angles, entry points)	Improved screw positioning accuracy and alignment; reproducible entry points and trajectories; reduced need for intraoperative adjustments; reduced fluoroscopy use in selected procedures	Requires stable anatomical landmarks; affected by soft-tissue injury and swelling; preparation time and heterogeneous clinical evidence	Moderate clinical evidence derived from small cohorts and heterogeneous anatomical indications
3D-printed patient-specific implants	Definitive reconstruction in complex defects (post-traumatic segmental bone loss, deformity, failed fixation)	Patient-specific implant geometry with porous titanium structures supporting implant integration and stability; favorable outcomes reported in small clinical series	Limited trauma-specific clinical data; manufacturing timelines; regulatory and certification requirements	Emerging clinical application supported by small clinical series and biomechanical studies
Temporary 3D-printed external fixation devices	Customizable temporary stabilization in staged trauma care	Potentially lower production cost and customizable designs; mechanical performance comparable to standard fixators in biomechanical studies	Evidence mainly limited to design and biomechanical feasibility studies; limited clinical validation	Early/experimental stage

## Data Availability

No new data were created or analyzed in this study. Data sharing is not applicable to this article.

## References

[1] Zhao Y., Wang Z., Zhao J., Hussain M., Wang M. (2022). Additive Manufacturing in Orthopedics: A Review. ACS Biomater. Sci. Eng.

[2] Fong F.J.Y., Onggo J.D., Munro Y.L., Yam M.G.J. (2025). Applications of 3D printing in orthopedics: A scoping review. Eur. J. Orthop. Surg. Traumatol..

[3] Prządka M., Pająk W., Kleinrok J., Pec J., Michno K., Karpiński R., Baj J. (2025). Advances in 3D Printing Applications for Personalized Orthopedic Surgery: From Anatomical Modeling to Patient-Specific Implants. J. Clin. Med..

[4] Levesque J.N., Shah A., Ekhtiari S., Yan J.R., Thornley P., Williams D.S. (2020). Three-dimensional printing in orthopaedic surgery: A scoping review. EFORT Open Rev..

[5] Bücking T.M., Hill E.R., Robertson J.L., Maneas E., Plumb A.A., Nikitichev D.I. (2017). From medical imaging data to 3D printed anatomical models. PLoS ONE.

[6] Meng M., Wang J., Sun T., Zhang W., Zhang J., Shu L., Li Z. (2022). Clinical applications and prospects of 3D printing guide templates in orthopaedics. J. Orthop. Transl..

[7] Wu C., Deng J., Li T., Tan L., Yuan D. (2020). Combined 3D Printed Template to Guide Iliosacral Screw Insertion for Sacral Fracture and Dislocation: A Retrospective Analysis. Orthop. Surg..

[8] Rong C., Zhang Q., Zhu S., Zhang G., Zeng J., Han Q., Guo Y. (2024). 3D printed guide-assisted percutaneous screw fixation for minimally displaced scaphoid waist fractures with delayed diagnosis or presentation. BMC Musculoskelet. Disord..

[9] Assink N., Oldhoff M.G.E., ten Duis K., Kraeima J., Doornberg J.N., Witjes M.J.H., de Vries J.-P.P.M., Meesters A.M.L., Ijpma F.F.A. (2024). Development of patient-specific osteosynthesis including 3D-printed drilling guides for medial tibial plateau fracture surgery. Eur. J. Trauma Emerg. Surg..

[10] Fragomen A.T., Rozbruch S.R. (2007). The Mechanics of External Fixation. HSS J. Musculoskelet. J. Hosp. Spec. Surg..

[11] Samaila E.M., Negri S., Zardini A., Bizzotto N., Maluta T., Rossignoli C., Magnan B. (2020). Value of three-dimensional printing of fractures in orthopaedic trauma surgery. J. Int. Med. Res..

[12] You W., Liu L.J., Chen H.X., Xiong J.Y., Wang D.M., Huang J.H., Ding J., Wang D. (2016). Application of 3D printing technology on the treatment of complex proximal humeral fractures (Neer3-part and 4-part) in old people. Orthop. Traumatol. Surg. Res..

[13] Bizzotto N., Sandri A., Regis D., Romani D., Tami I., Magnan B. (2015). Three-Dimensional Printing of Bone Fractures. Surg. Innov..

[14] Dust T., Henneberg J.E., Hartel M.J., Korthaus A., Ballhause T., von Rehlingen-Prinz F., Streckenbach A., Keller J., Frosch K.-H., Krause M. (2025). 3D printing improves preoperative decision making for patient positioning and surgical approach selection for tibial plateau fractures. Sci. Rep..

[15] Ozturk A.M., Suer O., Derin O., Ozer M.A., Govsa F., Aktuglu K. (2020). Surgical advantages of using 3D patient-specific models in high-energy tibial plateau fractures. Eur. J. Trauma Emerg. Surg..

[16] Si C., Bai B., Cong W., Zhang L., Guan R. (2024). Efficacy of 3D printing-assisted treatment for acetabular fractures. Jt. Dis. Relat. Surg..

[17] Morgan C., Khatri C., Hanna S.A., Ashrafian H., Sarraf K.M. (2019). Use of three-dimensional printing in preoperative planning in orthopaedic trauma surgery: A systematic review and meta-analysis. World J. Orthop..

[18] Mendonça C.J.A., Guimarães RMda R., Pontim C.E., Gasoto S.C., Setti J.A.P., Soni J.F., Schneider B. (2023). An Overview of 3D Anatomical Model Printing in Orthopedic Trauma Surgery. J. Multidiscip. Healthc..

[19] Sun L., Liu H., Xu C., Yan B., Yue H., Wang P. (2020). 3D printed navigation template-guided minimally invasive percutaneous plate osteosynthesis for distal femoral fracture: A retrospective cohort study. Injury.

[20] Chen L., Luo L., Zhang X., Li L., Liu D. (2025). Clinical efficacy analysis of anterior superior iliac spine 3D-printed guide plate-assisted sacroiliac screw placement for the treatment of pelvic fractures. Front. Surg..

[21] Pastor T., Nagy L., Fürnstahl P., Roner S., Pastor T., Schweizer A. (2022). Three-Dimensional Planning and Patient-Specific Instrumentation for the Fixation of Distal Radius Fractures. Medicina.

[22] Liang B., Chen Q., Liu S., Chen S., Yao Q., Wei B., Xu Y., Tang C., Wang L. (2020). A feasibility study of individual 3D-printed navigation template for the deep external fixator pin position on the iliac crest. BMC Musculoskelet. Disord..

[23] Dall’Ava L., Hothi H., Henckel J., di Laura A., Shearing P., Hart A. (2019). Comparative analysis of current 3D printed acetabular titanium implants. 3D Print. Med..

[24] McAnena A.P., McClennen T., Zheng H. (2025). Patient-Specific 3-Dimensional-Printed Orthopedic Implants and Surgical Devices Are Potential Alternatives to Conventional Technology But Require Additional Characterization. Clin. Orthop. Surg..

[25] Abar B., Kwon N., Allen N.B., Lau T., Johnson L.G., Gall K., Adams S.B. (2022). Outcomes of Surgical Reconstruction Using Custom 3D-Printed Porous Titanium Implants for Critical-Sized Bone Defects of the Foot and Ankle. Foot Ankle Int..

[26] MacLeod A., Patterson M., MacTear K., Gill H.S. (2020). 3D printed locking osteosynthesis screw threads have comparable strength to machined or hand-tapped screw threads. J. Orthop. Res..

[27] Liu Y., Shen J., Tang Y., Zhang Y., Ma H., Zhou Y. (2024). Comparison of Novel 3D-printed Stepped Porous Metal Cones and Metaphyseal Sleeves for Reconstruction of Severe Knee Bone Defects: Short-term Clinical Outcomes. Orthop. Surg..

[28] Bose D., Piper D. (2021). Temporary external fixation in the management of orthopaedic trauma. Orthop. Trauma.

[29] MacFadden L.N., Adams L.W., Boerhave C., O’Connor H.A., VanDerWolde B.K., Skelley N.W. (2024). Mechanical Analysis of a Novel 3D-printed External Fixator Design Versus Industry-standard External Fixators. J. Am. Acad. Orthop. Surg..

[30] Skelley N.W. (2023). Design and Development of a Novel 3-D Printed External Fixation Device for Fracture Stabilization. 3D Print. Med..

[31] Venter R., Kotze L., Ferreira N. (2022). A clinician-run 3D-printing laboratory for orthopaedic preoperative planning: An illustrative case series. SA Orthop. J..

[32] Long T., Tan L., Liu X. (2025). Three-dimensional printing in modern orthopedic trauma surgery: A comprehensive analysis of technical evolution and clinical translation. Front. Med..

[33] Daley B.J., Cecil W., Clarke C.P., Cofer J.B., Guillamondegui O.D. (2015). How Slow Is Too Slow? Correlation of Operative Time to Complications: An Analysis from the Tennessee Surgical Quality Collaborative. J. Am. Coll. Surg..

[34] Hess S., Husarek J., Müller M., Eberlein S.C., Klenke F.M., Hecker A. (2024). Applications and accuracy of 3D-printed surgical guides in traumatology and orthopaedic surgery: A systematic review and meta-analysis. J. Exp. Orthop..

[35] Kampkuiper N., ten Heggeler R., Nellensteijn J., Brusse-Keizer M., Tuijthof G., Koenrades M., Schröder F. (2025). Clinical added value of 3D printed patient-specific guides in orthopedic surgery (excluding knee arthroplasty): A systematic review. Arch. Orthop. Trauma Surg..

[36] Kermavnar T., Shannon A., O’Sullivan K.J., McCarthy C., Dunne C.P., O’Sullivan L.W. (2021). Three-Dimensional Printing of Medical Devices Used Directly to Treat Patients: A Systematic Review. 3D Print. Addit. Manuf..

[37] US Food and Drug Administration (2017). Technical Considerations for Additive Manufactured Medical Devices: Guidance for Industry and Food and Drug Administration Staff.

[38] Schick V.D., Zampogna B., Marrara G., Siracusano L., Larizza L., Calaciura S., Sanzarello I., Marinozzi A., Leonetti D. (2025). Custom-Made 3D-Printed Titanium Implants for Managing Segmental Distal Tibial Bone Defects: A Systematic Literature Review. J. Clin. Med..

[39] O’Connor H.A., Adams L.W., MacFadden L.N., Skelley N.W. (2023). 3D Printed Orthopaedic External Fixation Devices: A Systematic Review. 3D Print. Med..

[40] Verhey J.T., Haglin J.M., Verhey E.M., Hartigan D.E. (2020). Virtual, augmented, and mixed reality applications in orthopedic surgery. Int. J. Med. Robot..

[41] Karthik K., Colegate-Stone T., Dasgupta P., Tavakkolizadeh A., Sinha J. (2015). Robotic surgery in trauma and orthopaedics: A systematic review. Bone Jt. J..

